# Novel minimally invasive carpal tunnel release using a specialized surgical kit: a prospective multi-center case series

**DOI:** 10.1186/s12891-025-08612-0

**Published:** 2025-04-08

**Authors:** Hsuan-Fu Chen, Shun-Min Chang, Chih-Ming Kao, Yu-Lin Chen, Li-Ting Kao, Ya-Chuan Hsu, Yin-Chih Fu, Yan-Hsiung Wang, Wen-Chih Liu, Tien-Ching Lee

**Affiliations:** 1 Department of Orthopedics, Division of Sports Medicine and Orthopaedic Trauma, Kaohsiung Medical University Hospital, Kaohsiung Medical University, No.100, Tzyou 1 Road, Kaohsiung, 80756 Taiwan; 2 Orthopaedic Research Center, College of Medicine, Kaohsiung Medical University Hospital, Kaohsiung Medical University, Kaohsiung, 80708 Taiwan; 3 Department of Orthopedics, Kaohsiung Municipal Siaogang Hospital, Kaohsiung, 81266 Taiwan; 4 Regenerative Medicine and Cell Therapy Research Center, Kaohsiung Medical University, Kaohsiung, 80708 Taiwan; 5 Department of Orthopedics, China Medical University Hospital, Taichung, 404327 Taiwan; 6 Graduate Institute of Medicine, College of Medicine, Kaohsiung Medical University, Kaohsiung, 807 Taiwan; 7 Department of Nursing, Kaohsiung Medical University Hospital, Kaohsiung Medical University, Kaohsiung, 80756 Taiwan; 8 Department of Orthopedics, College of Medicine, Kaohsiung Medical University, Kaohsiung, 807378 Taiwan; 9 School of Dentistry, College of Dental Medicine, Kaohsiung Medical University, Kaohsiung, 807378 Taiwan; 10 Department of Medical Research, Kaohsiung Medical University Hospital, Kaohsiung, 80756 Taiwan

**Keywords:** Carpal tunnel syndrome, Local anesthetic, Minimally invasive surgery, Nerve conduction velocity, Pinch and grip strengths

## Abstract

**Background:**

Carpal tunnel syndrome (CTS) is the most common peripheral nerve compression neuropathy. While established surgical techniques have demonstrated reliable outcomes and safety profiles, innovations in minimally invasive approached continue to emerge. This study evaluates a novel minimally invasive surgical technique using a specialized instrument for carpal tunnel release.

**Methods:**

In this prospective multi-center case series, 41 patients underwent minimally invasive carpal tunnel release using a novel surgical kit. Outcomes were assessed through Visual Analog Scale (VAS), Boston Carpal Tunnel Questionnaire (BCTQ), grip and pinch strength measurements, and nerve conduction velocity (NCV) testing at regular intervals over 24 weeks post-surgery.

**Results:**

Mean surgical time was 7.02 min. Significant improvements were observed in VAS scores (LS-Mean − 0.57, *P* < 0.0001) and BCTQ scores (Symptom Severity: LS-Mean − 2.62, *P* < 0.0001; Functional Status: LS-Mean − 1.20, *P* < 0.0001) by 24 weeks. Grip and pinch strengths showed significant improvement from 2 weeks post-surgery. Mean time to return to work was 18.2 days. NCV testing demonstrated significant improvements in both latency (LS-Mean − 0.57, *P* < 0.0001) and velocity (LS-Mean 5.79, *P* < 0.0001). One superficial infection and two cases of temporary numbness were reported, with no recurrent CTS observed.

**Conclusions:**

This novel minimally invasive technique demonstrates promising clinical outcomes with shortened operative time, rapid symptom relief, and early functional recovery. While larger randomized studies are needed, these preliminary findings suggest this technique may be a valuable addition to current surgical options for CTS.

**Trial registration:**

Clinicaltrials.gov, NCT05067205. Prospectively registered, date of first registration: 05/10/2021 (https://clinicaltrials.gov/study/NCT05067205).

**Supplementary Information:**

The online version contains supplementary material available at 10.1186/s12891-025-08612-0.

## Background

Carpal tunnel syndrome (CTS), which is caused by the entrapment of the median nerve at the wrist, is the most common peripheral nerve compressive neuropathy globally [1]. The prevalence of CTS in the general population is 1%–5% with a higher prevalence rate among women than men [2–4]. CTS is more prevalent among the working population due to repetitive and inappropriate use of the hands, carrying heavy loads, and hand exposure to vibration or high pressure [3–5] reducing work time and efficiency. Therefore, CTS contributes to occupational disability [6].

Patients with CTS initially receive conservative management such as immobilization, rehabilitation, and nonsteroidal anti-inflammatory drugs. When conservative treatments fail to alleviate CTS symptoms, surgical intervention is recommended. The gold standard surgical approach is traditional open carpal tunnel release (OCTR), a proven effective procedure with fewer complications. However, OCTR may be accompanied by wound problems and requires a longer recovery time [1]. Previous instruments for carpal tunnel release, such as the Paine retinaculotome [7] and the Indiana Tome [8], have laid the groundwork for advanced methodologies in surgical decompression. Menon J. and Resnick CT et al. provided insights into endoscopic carpal tunnel release (ECTR) with notable outcomes [9, 10].

Despite these advancements, current techniques continue to grapple with balancing procedural safety, and efficiency, while ensuring minimal patient discomfort and recovery time. This study proposes a novel surgical kit designed to address these concerns through a minimally invasive approach.

## Methods

### Study design and participants

This prospective, multi-center case series was conducted at two university-affiliated hospitals in Taiwan. We enrolled 41 patients with CTS who met both the CTS- 6 diagnostic criteria [11] and showed positive nerve conduction velocity (NCV) findings. All surgical procedures were performed by two experienced hand surgeons using a novel MICTR technique between September 2021 and December 2022. Inclusion criteria consisted of patients aged 20 years or older who had both clinical and electrodiagnostic confirmation of CTS and had failed conservative treatment for at least three months. Exclusion criteria included recurrent CTS, concurrent cervical radiculopathy, history of systemic immunosuppressive or glucocorticosteroid therapy, and diabetes mellitus. The study protocol was approved by the Institutional Review Board (KMUHIRB-F(I)− 20,200,063).

### The design of surgical device

**Fig. 1:**
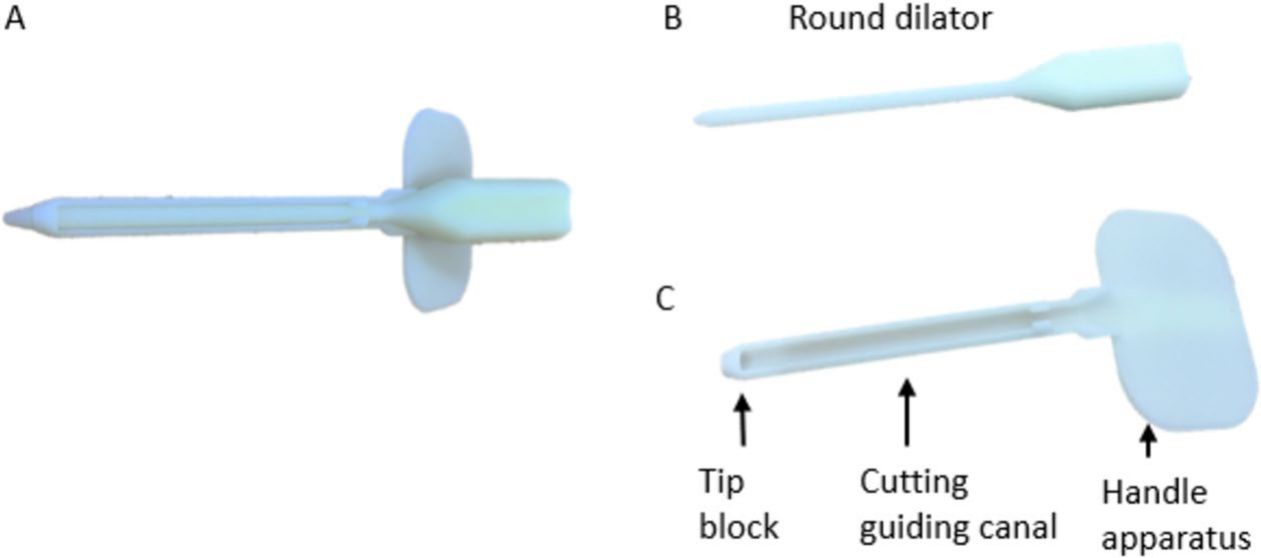
Components of the novel minimally invasive carpal tunnel release surgical kit. **A** Complete assembly of the CTS mini kit. **B** Round dilator for initial tunnel dilation. **C** Guiding device featuring a protective tip block, cutting guide canal, and ergonomic handle for precise control during release

The instrument was designed to incorporate the technical advantages of the current MICTR. By means of the invented instrument, we could perform the carpal tunnel release with a minimal incision and fewer surgical instrument to achieve better and faster recovery. This invented instrument consisted of two components: the round dilator and the guiding device (Fig. 1). The first part of the instrument consisted of a round dilator, which increased the surgical space. The second part of the instrument, the guiding device, comprised three distinct elements: a tip block to alert the surgeon to the depth of release, a cutting guiding canal to ensure a precise cutting pathway without violating other structures, and a handle apparatus designed for ease of use during the surgery. The surgical kit is designed as a single-use device, manufactured from medical-grade stainless steel for the cutting components and sterile-grade plastic for the handling portions. All materials comply with medical device safety standards.

### Surgical technique

All procedures were performed by two experienced surgeons (Y.-C. F. and T.-C. L) under local anesthesia using the following protocol:

- Pre-operative Preparation:
  - Upper arm tourniquet placement and limb exsanguination (except in patients with arteriovenous fistulas for hemodialysis)
  - Administration of local anesthesia (2% lidocaine HCl, Xylocaine, 20 mL/vial) at the mid-proximal wrist crease

**Fig. 2:**
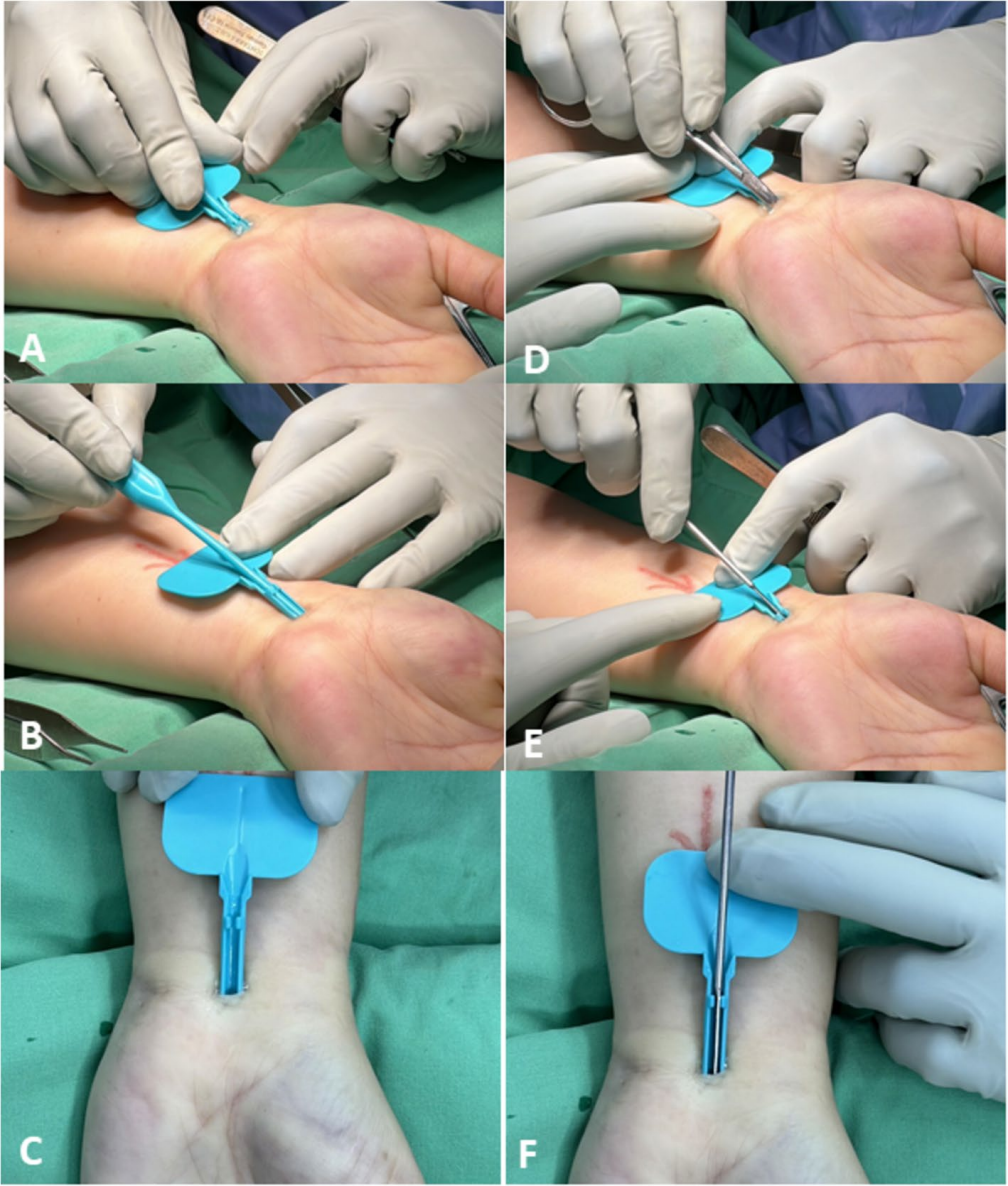
Step-by-step surgical technique for minimally invasive carpal tunnel release. **A** Creation of a 1-cm transverse skin incision at the proximal wrist crease. **B** Blunt dissection using Stevens Tenotomy Scissors under loupe magnification to expose the flexor retinaculum. **C** Application of 2% lidocaine HCl gel between the flexor retinaculum and flexor tendon. **D** Placement of the novel surgical instrument beneath the flexor retinaculum. **E** Safety verification with mosquito forceps to ensure clear cutting pathway. **F** Complete transection of the flexor retinaculum using scalpel

Surgical Technique with Sequential Imaging:

1. Initial Approach
  Creation of a 1-cm transverse skin incision at proximal wrist crease (Fig. 2A)
  Blunt dissection using Stevens Tenotomy Scissors under loupe magnification to identify flexor retinaculum (Fig. 2B)
  Introduction of 2% lidocaine HCl gel (Xylocaine jelly, 3 mL) between flexor retinaculum and flexor tendon (Fig. 2C)
2. Instrument Placement and Release
  Insertion of novel surgical instrument beneath flexor retinaculum (Fig. 2D)
  Careful removal of round dilator
  Safety verification using mosquito forceps to ensure no structures adhere to flexor retinaculum along intended cutting pathway (Fig. 2E)
  Complete carpal tunnel release using scalpel (Fig. 2F)
  Confirmation of successful release through easy dilator insertion
3. Wound Closure and Post-Release Care
  Closure with 4 - 0 nylon sutures
  Tourniquet deflation
  Five-minute local compression for hemostasis

### Clinical follow-up

Patients were evaluated at the Orthopedic Outpatient Department (OPD) on postoperative day 3 and at weeks 2, 4, 16, and 24. Immediate post-surgical care included 30-min direct wound compression and ice pack application to prevent hematoma formation. Acetaminophen (Depyretin 500 mg/tablet) was routinely prescribed for initial pain management for 3 days.

At the first follow-up (day 3), assessments included Visual Analog Scale (VAS), grip and pinch strength measurements, and wound examination. Acetaminophen was continued until the 2-week follow-up. At week 2, surgical outcomes were evaluated, sutures were removed if wound healing was satisfactory, and pain management was switched to topical etofenamate (Tonin Gel 40 g/tube). Additionally, patients received fursultiamine (B1 derivative) 50 mg + riboflavin (B2) 5 mg (Alinamin F 50 mg/tablet) for two weeks. Subsequent follow-up visits included comprehensive outcome assessments as detailed below.

### Study objectives and outcome measures

This prospective multi-center study aimed to evaluate the safety and efficacy of a novel minimally invasive surgical technique for carpal tunnel release using a specialized surgical kit. The primary endpoints were changes in symptom severity and functional status as measured by the Boston Carpal Tunnel Questionnaire (BCTQ) at 24 weeks post-surgery, along with safety outcomes through the follow-up period. Secondary endpoints included changes in pain severity, recovery of hand function, improvement in nerve conduction parameters, procedural efficiency metrics, and patient satisfaction.

Clinical outcomes were evaluated preoperatively and at scheduled follow-up visits on day 3 and at weeks 2, 4, 16, and 24. Pain assessment was conducted using the Visual Analog Scale (VAS). The BCTQ, administered using its validated Chinese version for our Taiwanese patient population [13], comprised two components: the Symptom Severity Scale (BCTQ-S) consisting of 11 items scored from 1 (normal) to 5 (severe), and the Functional Status Scale (BCTQ-F) evaluating 8 daily activities scored from 1 (no difficulty) to 5 (cannot perform) [12].

Functional recovery was assessed through grip and pinch strength measurements. Nerve conduction velocity (NCV) testing was performed at baseline and 24 weeks postoperatively to evaluate neurophysiological improvement. Throughout the follow-up period, we monitored for complications including pillar pain, persistent numbness, and surgical site infection. Additional outcome measures included surgical time, duration until return to work, and patient satisfaction assessed at the final 24-week follow-up visit.

### Statistical analysis

Quantitative variables were described using means, standard deviations (SD), medians, and interquartile range (IQR) for age, body mass index (BMI), surgical time, time to return to work, and satisfaction. Qualitative variables were described for the study sample using frequencies and percentages. Least square mean (LS-Mean), standard errors, and confidence interval (CI) were calculated for the mean change of pinch and grip strength, BCTQ scores, and NCV from baseline to each follow-up visit. All tests were two-tailed, with a significance level (P) defined as *P* of < 0.05. IBM®-SPSS® version 20.0 for Windows® was used for statistical analysis.

### Technique patents

The instrument had been granted several patents from Taiwan (invention patent number: I649061), China (invention patent number: 201780064017.5), the United States (invention patent number: 16/346,107), Europe (invention patent number: 17863950.6), and Singapore (invention patent number: 11201903751U).

## Results

### Study population

A total of 41 patients (11 men, 30 women) with a mean age of 59.78 ± 11.32 years underwent MICTR using the novel instrument. The majority of patients were nonsmokers (92.7%) and presented with positive Tinel's sign (70.7%) preoperatively. The cohort had a mean BMI of 26.07 ± 4.91 and a median BMI of 24.9 (IQR: 23.2–29.4), classifying them as overweight according to World Health Organization criteria.

Follow-up compliance was high in the early postoperative period, with all patients attending scheduled visits at day 3 and weeks 2 and 4. At week 16, 32 patients (78.0%) completed follow-up visits, while 37 patients (90.2%) attended the final 24-week follow-up. The four patients who missed the final visit reported complete symptom resolution as their reason for non-attendance. Three patients neither returned to work nor provided satisfaction feedback at the 24-week follow-up (Table 1 and Supplement Table 5).

**Table 1:** Demographic and clinical characteristics of patients undergoing novel minimally invasive carpal tunnel release

Item, Unit, and Case Number (n)	Value^a^
Age, year, *n* = 41	
Mean (SD)	59.78 (11.32)
Median (IQR)	57.0 (53.0–65.0)
Hospital, n (%), *n* = 41	
KMTTH^b^	15 (36.6)
KMUH^c^	25 (61.0)
KMTTH and KMUH	1 (2.4)
Sex, n (%), *n* = 41	
Males	11 (26.8)
Females	30 (73.2)
Height (cm), *n* = 41	
Mean (SD)	159.61 (7.01)
Median (IQR)	159.0 (155.0–164.0)
Weight (kg), *n* = 41	
Mean (SD)	66.92 (16.27)
Median (IQR)	63.0 (56.0–77.0)
BMI (kg/m^2^), *n* = 41	
Mean (SD)	26.07 (4.91)
Median (IQR)	24.9 (23.2–29.4)
Smoker, n (%), *n* = 41	
No	38 (92.7)
Yes	3 (7.3)
Tinel’s sign, n (%), *n* = 41	
No	12 (29.3)
Yes	29 (70.7)
Surgical time(mins), Median (IQR), *n* = 41	
Mean (SD)	7.02 (2.30)
Median (IQR)	6.0 (5.5–8.0)
Return to work(days), 24 W, Median (IQR), *n* = 34	
Mean (SD)	18.18 (29.20)
Median (IQR)	7.0 (1.0–28.0)
Satisfaction, 24 W, Median (IQR), *n* = 34	
Mean (SD)	8.99 (1.91)
Median (IQR)	10.0 (8.0–10.0)

^a^Values are presented as mean (standard deviation [SD]), median (interquartile range [IQR] [Q1: 25% and Q3: 75%]), or percentage (%)

^b^Kaohsiung Municipal Ta-Tung Hospital

^c^Kaohsiung Medical University Hospital

### Primary outcomes

#### Symptom severity and functional status

**Fig. 3:**
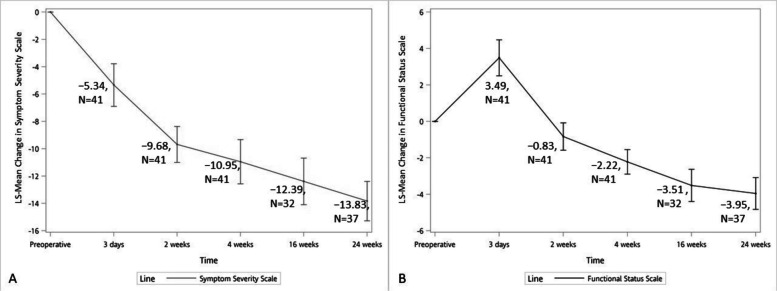
Changes in Boston Carpal Tunnel Questionnaire (BCTQ) scores following minimally invasive carpal tunnel release. **A** Mean change in symptom severity scale (BCTQ-S) from baseline through 24-week follow-up. **B** Mean change in functional status scale (BCTQ-F) from baseline through 24-week follow-up

The BCTQ showed significant improvements in both components at 24 weeks post-surgery. The symptom severity scale (BCTQ-S) demonstrated a significant reduction (LS-Mean − 2.62, 95% CI: − 3.13 to − 2.10, *P* < 0.0001), with continuous improvement throughout the follow-up period. The functional status scale (BCTQ-F) also showed significant improvement (LS-Mean − 1.20, 95% CI: − 1.57 to − 0.84, *P* < 0.0001), though with initial worsening at day 3 followed by steady improvement (Fig. 3A and B, Supplement Tables 4 and 5).

#### Safety outcomes and complications

Among the 41 patients, one developed a superficial surgical site infection that resolved after one week of oral cephradine (Unifradine 250 mg/capsule) without further sequelae. Two patients reported persistent numbness at 16 weeks postoperatively. In these two cases, perineural scarring or traction neuropathy were considered as potential causes. At the final follow-up, only one patient reported residual mild numbness, though notably improved compared to the preoperative state. Serial two-point discrimination testing showed no significant deterioration in sensory function at any follow-up visit. When revision surgery was discussed, this patient declined further intervention as the residual symptoms did not impact daily activities. No patients experienced recurrent CTS or required reoperation during the follow-up period.

### Secondary outcomes

#### Pain assessment

**Fig. 4:**
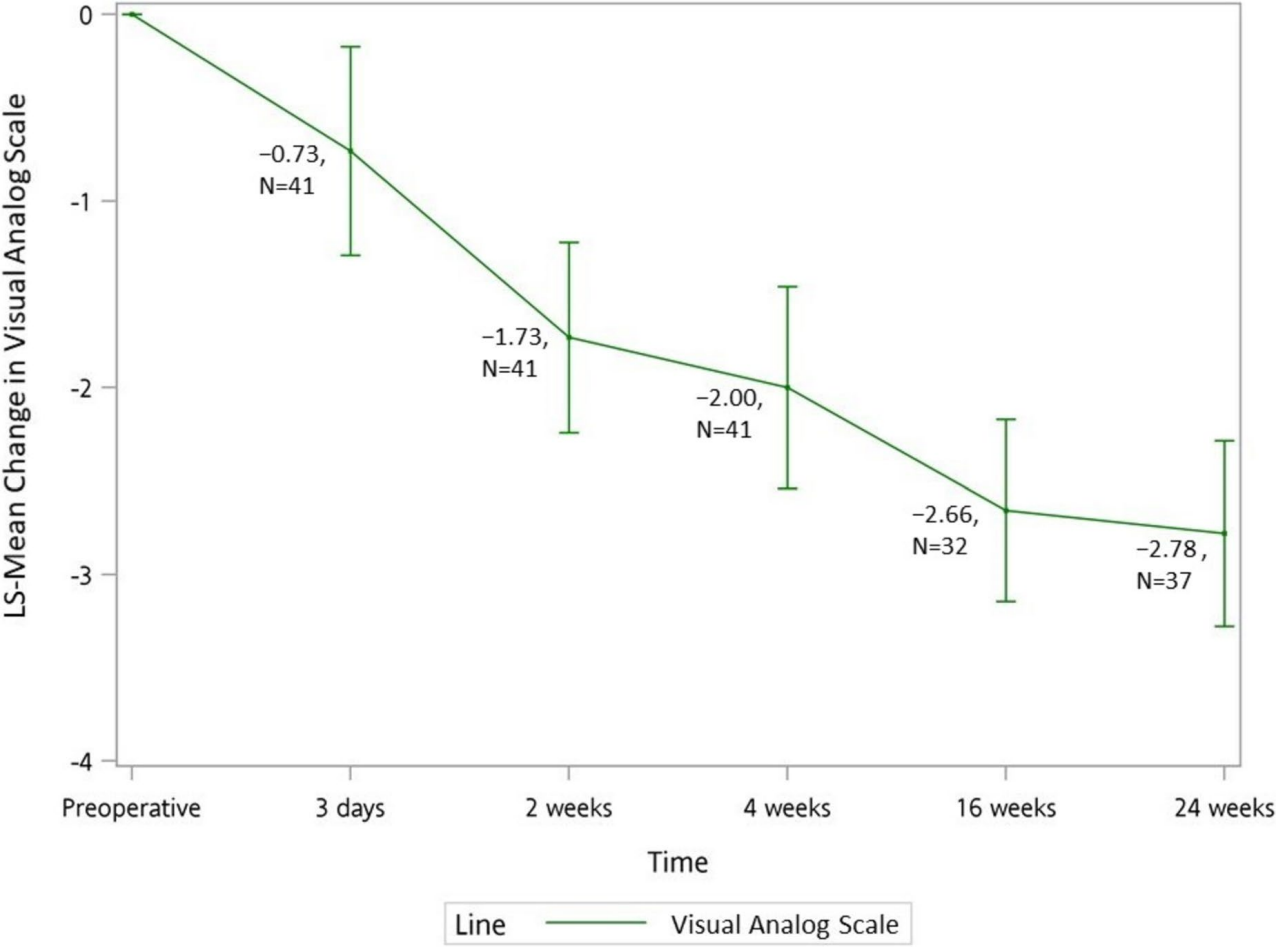
Changes in pain severity following minimally invasive carpal tunnel release as measured by Visual Analog Scale (VAS). Mean change from baseline through 24-week follow-up is shown

VAS scores showed significant reduction at 24 weeks (LS-Mean − 0.57, 95% CI: − 0.73 to − 0.41, *p* < 0.0001), with consistent improvement throughout follow-up (Fig. 4, Supplement Tables 1 and 5).

#### Functional recovery

**Fig. 5:**
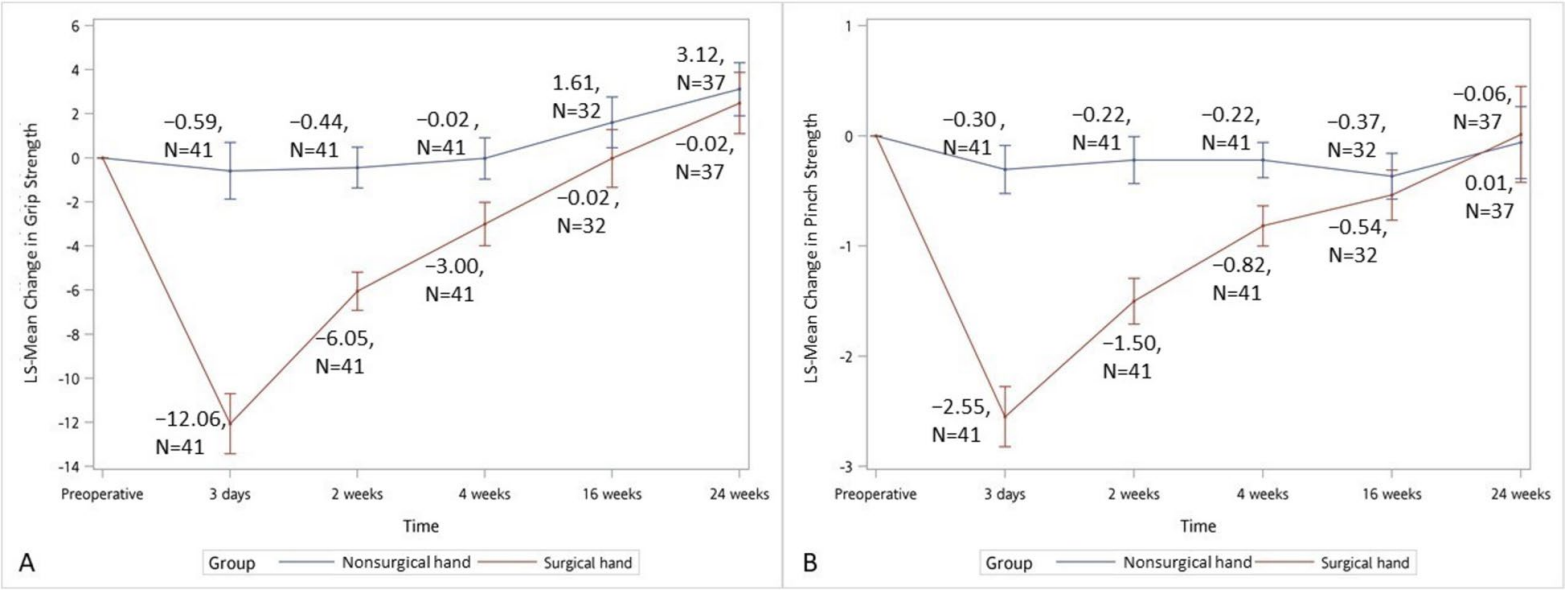
Changes in hand strength measurements following minimally invasive carpal tunnel release. **A** Mean change in grip strength from baseline through 24-week follow-up. **B** Mean change in pinch strength from baseline through 24-week follow-up

Grip strength (LS-Mean − 1.47, 95% CI: 0.91–2.04, *p* < 0.0001) and pinch strength (LS-Mean: 0.19, 95% CI: 0.04–0.35, *p* = 0.0134) showed significant improvements at 24 weeks, despite initial decrease at day 3 post-surgery (Fig. 5A and B, Supplement Tables 2, 3 and 5).

#### Neurophysiological mmprovement

Nerve conduction studies at 24 weeks showed significant improvements in the surgical hand for both latency (LS-Mean − 0.57; 95% CI − 0.83 to − 0.32, *P* < 0.0001) and velocity (LS-Mean 5.79; 95% CI 3.63 to 7.95, *P* < 0.0001). Comparison with the nonsurgical hand showed significant differences in both parameters (Table 2).

**Table 2:** Comparative analysis of nerve conduction parameters between surgical and nonsurgical hands at 24-week follow-up

	Surgical hand				Nonsurgical hand				Surgical vs nonsurgical hand	
	Presurgical	24 weeks post-surgery	Change from Baseline LS-Mean^a^ (95% CI^b^)	*P*-value	Presurgical	24 weeks post-surgery	Change from Baseline LS-Mean (95% CI)	*P*-value	Difference of LS-Mean (SE^c^; 95% CI)	*P*-value
Latency (ms)	4.63 (0.18)	4.05 (0.17)	− 0.57 (− 0.83 to − 0.32)	< 0.0001	3.98 (0.17)	3.94 (0.16)	− 0.04 (− 0.24 to 0.16)	0.6848	− 0.53 (− 0.84 to − 0.23)	0.0006
Velocity (m/s)	32.91 (1.30)	38.70 (1.42)	5.79 (3.63 to 7.95)	< 0.0001	39.32 (1.51)	40.62 (1.45)	1.30 (− 1.00 to 3.6)	0.2674	4.49 (2.13 to 6.85)	0.0002

^*^^a^*LS-Mean *least squares mean, ^b^CI confidence interval, ^c^*SE* standard error

#### Procedural efficiency

The mean surgical time was 7.02 min (SD: 2.30), with a median of 6.0 min (IQR 5.5–8.0). All procedures were performed under local anesthesia. Among 34 patients with complete data, mean time to return to work was 18.2 days (SD: 29.20) (Table 1).

#### Patient satisfaction

The mean satisfaction score at 24 weeks was 8.99 out of 10 (SD: 1.91), indicating high patient satisfaction with the procedure (Table 1).

## Discussion

### Primary endpoints

#### Symptom severity and functional status

Our study demonstrated significant improvements in both primary endpoints as measured by the Boston Carpal Tunnel Questionnaire. The symptom severity scale (BCTQ-S) showed marked improvement from day 3 post-surgery (LS-Mean − 2.62, *P* < 0.0001). Previous studies have shown similar improvements in BCTQ-S, though typically reporting changes from 1–2 weeks post-surgery [24, 25]. The functional status scale (BCTQ-F) demonstrated significant enhancement from week 2 onwards (LS-Mean − 1.20, *P* < 0.0001), with initial worsening at day 3. While other studies have reported improvements in both BCTQ-S and BCTQ-F from 1–2 weeks post-surgery [24, 25], few have examined hand function within the first week, making our early assessment particularly valuable.

#### Safety outcomes

The safety profile was favorable, with only three complications (7.3%) among 41 patients. This compares favorably with both OCTR and ECTR, where complication rates vary between 1–25% in the literature [14-21] Our low complication rate can be attributed to the instrument's unique design features, including a U-shaped cutting tunnel with a protective baffle, which differentiates it from traditional instruments and other minimally invasive approaches [22, 23].

### Secondary outcomes

#### Pain and functional recovery

VAS scores showed significant improvement (LS-Mean − 0.57, P < 0.0001) from day 3 post-surgery. Grip and pinch strength demonstrated significant recovery from week 2, reaching pre-surgical levels by week 16 and exceeding baseline by week 24. While José Dinis Carmo [24] and Kok Kheng Teh [27] reported strength recovery to presurgical conditions by 3 months using similar MICTR techniques, our timeline still represents improvement over traditional OCTR recovery periods [26].

#### Procedural efficiency

Our average surgical time of 7 min represents a notable improvement over both OCTR (10–25 min) and ECTR (13–21 min) reported in Koong et al.'s meta-analysis [14]. Even compared to similar MICTR devices, such as that used by José Dinis Carmo [24] with an 11-min average, our technique demonstrates enhanced efficiency. The mean return-to-work time of 18.2 days aligns with ECTR outcomes (12.1–18 days) and improves upon OCTR (21.2–26 days) [14].

#### Technical innovations and comparative analysis

While acknowledging that minimally invasive carpal tunnel release isn't new [28], our approach offers specific advantages over existing techniques. Unlike standard OCTR with its 2–4 cm incision, or other MICTR variations using Hook Knives [22] or modified retractors [23], our instrument incorporates specific design improvements for enhanced safety and minimal tissue damage. The specialized guiding canal and tip block represent advances over traditional instruments, potentially explaining our favorable outcomes. A systematic review by Koong et al. [14] found similar effectiveness between OCTR and ECTR in symptom alleviation. While Sayegh et al. [18] reported ECTR advantages in reducing morbidity and recovery time, Barnes et al. [6] noted its higher costs. Our technique appears to combine the benefits of both approaches—the safety of OCTR with the efficiency of ECTR—while potentially offering cost advantages through simplified instrumentation and shorter operative times.

## Limitations

This study has several limitations. First, although initially designed as a randomized controlled trial, recruitment challenges for the OCTR group led to modification to a single-arm study, preventing direct comparative assessment against conventional approaches. Second, while carpal tunnel syndrome is a common condition, our sample size of 41 patients, though sufficient for initial safety and efficacy evaluation, may be considered modest for definitive conclusions. Third, the 24-week follow-up period may be insufficient to detect long-term complications or adverse events.

Regarding technique-specific limitations, MICTR presents several challenges that warrant consideration [22–24]. The restricted visualization during the procedure may affect surgical precision and requires specific expertise. Complete ligament release must be carefully confirmed through indirect means, and the technique may not be suitable for all cases, particularly those with significant anatomical variations or concurrent pathologies requiring more extensive exposure. These limitations underscore the importance of proper patient selection and surgeon experience in achieving optimal outcomes. Additionally, while our results demonstrated favorable outcomes, all procedures were performed by two experienced hand surgeons, raising questions about outcome generalizability when the technique is adopted by surgeons with varying expertise levels. The learning curve for this technique, though potentially shorter than other minimally invasive approaches, requires further investigation.

Some authors (Y.-C. H., Y.-C. F., Y.-H. W., T.-C. L.) hold the patent for the surgical kit used in this study, presenting a potential conflict of interest. However, outcome assessments were conducted independently by non-patent-holding authors, and NCV measurements provided objective data to minimize potential bias. Despite reporting symptom improvement, four patients were lost to final follow-up, potentially affecting outcome assessment.

Future studies should address these limitations through larger multicenter trials comparing this technique with conventional approaches, extended follow-up periods to evaluate long-term outcomes, investigation of the learning curve across surgeons with varying experience levels, and improved strategies for patient retention during follow-up.

## Conclusions

Our novel MICTR technique demonstrates promising clinical outcomes with shortened operative time, rapid symptom relief, and early functional recovery in this initial cohort. The specialized surgical kit, incorporating safety features and standardized cutting guidance, achieved favorable results in our series. However, several important limitations should be considered: the single-arm study design precluded direct comparison with conventional techniques, the sample size was modest, and all procedures were performed by experienced surgeons, which may affect generalizability. While these preliminary findings suggest potential advantages, larger randomized controlled trials with diverse surgical expertise levels are needed to definitively establish comparative efficacy and safety. Future studies should also address the learning curve, long-term outcomes, and cost-effectiveness once commercialized. This technique may represent a valuable addition to current surgical options for carpal tunnel release, pending further validation through more comprehensive comparative studies.

## Supplementary Information

Additional file 1

## Data Availability

Data is provided within the manuscript and supplementary information files.

## Abbreviations

BCTS-S: Boston Carpal Tunnel Questionnaire-symptom severity scale
BCTS-F: Boston Carpal Tunnel Questionnaire-functional status scale
BMI: Body mass index
CTR: Carpal tunnel release
CTS: Carpal tunnel syndrome
CI: Confidence intervals
ECTR: Endoscopic Carpal tunnel release
IQR: Interquartile range
KMUH: Kaohsiung Medical University Hospital
KMUTTH: Kaohsiung Municipal Ta-Tung Hospital
LS-Mean: Least square mean
MICTR: Minimal-invasive carpal tunnel release
NCV: Nerve conduction velocity
OCTR: Open Carpal tunnel release
OPD: Outpatient Department
SD: Standard deviation
VAS: Visual Analog Scale
