# Supplementary material for: Novel minimally invasive carpal tunnel release using a specialized surgical kit: a prospective multi-center case series
Source: BMC Musculoskelet Disord. 2025 Apr 8;26:346. doi: 10.1186/s12891-025-08612-0 (PMC11980155; doi:10.1186/s12891-025-08612-0)
Supplement: Supplementary file 1 — Additional file 1 [file 12891_2025_8612_MOESM1_ESM.zip › Supplement Table 1.docx]

Supplement Table1. Time course of mean change in VAS from baseline, 3 days, and 2, 4, 16, and 24 weeks

|  | LS-Means (SE) | Chang from Baseline  LS-Means (95% CI) | P value | Chang from Baseline  LS-Means (95% CI) | P value | Chang from Baseline  LS-Means (95% CI) | P value | Chang from Baseline  LS-Means (95% CI) | P value | Chang from Baseline  LS-Means (95% CI) | P value |
| --- | --- | --- | --- | --- | --- | --- | --- | --- | --- | --- | --- |
| VAS score |  |  |  |  |  |  |  |  |  |  |  |
| Preoperative | 3.24 (0.46) | **Reference** |  |  |  |  |  |  |  |  |  |
| 3d | 2.51 (0.41) | -0.73 (-1.83 to 0.36) | 0.1909 | **Reference** |  |  |  |  |  |  |  |
| 2w | 1.51 (0.28) | -1.73 (-2.73 to -0.73) | 0.0007 | -1.00 (-1.70 to -0.30) | 0.0051 | **Reference** |  |  |  |  |  |
| 4w | 1.24 (0.28) | -2.00 (-3.06 to -0.94) | 0.0002 | -1.27 (-2.00 to -0.54) | 0.0007 | -0.27 (-0.69 to 0.15) | 0.2072 | **Reference** |  |  |  |
| 16w | 0.59 (0.23) | -2.66 (-3.61 to -1.70) | <0.0001 | -1.93 (-2.69 to -1.16) | <0.0001 | -0.93 (-1.49 to -0.37) | 0.0012 | -0.66 (-1.13 to -0.18) | 0.0067 | **Reference** |  |
| 24w | 0.46 (0.17) | -2.78 (-3.75 to -1.81) | <0.0001 | -2.05 (-2.90 to -1.20) | <0.0001 | -1.05 (-1.58 to -0.52) | 0.0001 | -0.78 (-1.36 to -0.20) | 0.0088 | -0.12 (-0.68 to 0.44) | 0.6708 |
| Preoperative to 24w |  | -0.57 (-0.73 to -0.41) | <0.0001 |  |  |  |  |  |  |  |  |

LS-Mean: least squares mean; SE: standard error; CI: confidence intervals.
