# Supplementary material for: Novel minimally invasive carpal tunnel release using a specialized surgical kit: a prospective multi-center case series
Source: BMC Musculoskelet Disord. 2025 Apr 8;26:346. doi: 10.1186/s12891-025-08612-0 (PMC11980155; doi:10.1186/s12891-025-08612-0)
Supplement: Supplementary file 1 — Additional file 1 [file 12891_2025_8612_MOESM1_ESM.zip › Supplement Table 2.docx]

Supplement Table2. Time course of mean change in grip strength from baseline, 3 days, and 2, 4, 16, and 24 weeks

|  | LS-Mean (SE) | Change from Baseline  LS-Mean (95% CI) | *P*-value | Change from Baseline  LS-Mean (95% CI) | *P*-value | Change from Baseline  LS-Mean (95% CI) | *P*-value | Change from Baseline  LS-Mean (95% CI) | *P*-value | Change from Baseline  LS-Mean (95% CI) | *P*-value |
| --- | --- | --- | --- | --- | --- | --- | --- | --- | --- | --- | --- |
| Non-Surgical hand |  |  |  |  |  |  |  |  |  |  |  |
| Presurgical | 22.59 (1.63) | **Reference** |  |  |  |  |  |  |  |  |  |
| 3 days | 22.00 (1.60) | −0.59 (−3.11 to 1.94) | 0.6490 | **Reference** |  |  |  |  |  |  |  |
| 2 weeks | 22.15 (1.22) | −0.44 (−2.25 to 1.37) | 0.6341 | 0.15 (−1.84 to 2.13) | 0.8851 | **Reference** |  |  |  |  |  |
| 4 weeks | 22.56 (1.46) | −0.02 (−1.87 to 1.82) | 0.9794 | 0.56 (−1.23 to 2.35) | 0.5399 | 0.41 (−1.25 to 2.07) | 0.6245 | **Reference** |  |  |  |
| 16 weeks | 24.20 (1.54) | 1.61 (−0.65 to 3.87) | 0.1620 | 2.20 (0.12 to 4.27) | 0.0385 | 2.05 (0.30 to 3.80) | 0.0215 | 1.63 (−0.18 to 3.45) | 0.0778 | **Reference** |  |
| 24 weeks | 25.71 (1.61) | 3.12 (0.76 to 5.48) | 0.0096 | 3.71 (1.09 to 6.32) | 0.0055 | 3.56 (1.34 to 5.78) | 0.0017 | 3.15 (0.91 to 5.39) | 0.0059 | 1.51 (−0.04 to 3.07) | 0.0567 |
| Presurgical to 24 weeks |  | 0.65 (0.22 to 1.08) | 0.0032 |  |  |  |  |  |  |  |  |
| Surgical hand |  |  |  |  |  |  |  |  |  |  |  |
| Presurgical | 19.85 (1.63) | **Reference** |  |  |  |  |  |  |  |  |  |
| 3 days | 7.79 (1.03) | −12.06 (−14.72 to −9.40) | <0.0001 | **Reference** |  |  |  |  |  |  |  |
| 2 weeks | 13.80 (1.36) | −6.05 (−7.75 to −4.35) | <0.0001 | 6.01 (3.89 to 8.14) | <0.0001 | **Reference** |  |  |  |  |  |
| 4 weeks | 16.85 (1.61) | −3.00 (−4.93 to −1.07) | 0.0024 | 9.06 (6.76 to 11.37) | <0.0001 | 3.05 (1.50 to 4.60) | 0.0001 | **Reference** |  |  |  |
| 16 weeks | 19.83 (1.70) | −0.02 (−2.60 to 2.55) | 0.9852 | 12.04 (9.20 to 14.87) | <0.0001 | 6.02 (3.70 to 8.35) | <0.0001 | 2.98 (1.19 to 4.76) | 0.0011 | **Reference** |  |
| 24 weeks | 22.34 (1.75) | 2.49 (−0.24 to 5.21) | 0.0738 | 14.55 (11.30 to 17.80) | <0.0001 | 8.54 (5.94 to 11.13) | <0.0001 | 5.49 (3.14 to 7.83) | <0.0001 | 2.51 (0.87 to 4.16) | 0.0027 |
| Presurgical to 24 weeks | 22.59 (1.63) | 1.47 (0.91 to 2.04) | <0.0001 |  |  |  |  |  |  |  |  |

LS-Mean: least squares mean; SE: standard error; CI: confidence intervals.
