# Supplementary material for: Novel minimally invasive carpal tunnel release using a specialized surgical kit: a prospective multi-center case series
Source: BMC Musculoskelet Disord. 2025 Apr 8;26:346. doi: 10.1186/s12891-025-08612-0 (PMC11980155; doi:10.1186/s12891-025-08612-0)
Supplement: Supplementary file 1 — Additional file 1 [file 12891_2025_8612_MOESM1_ESM.zip › Supplement Table 3.docx]

Supplement Table 3. Time course of mean change in pinch strength from baseline, 3 days, and 2, 4, 16, and 24 weeks

|  | LS-Mean (SE) | Change from Baseline  LS-Mean (95% CI) | *P*-value | Change from Baseline  LS-Mean (95% CI) | *P*-value | Change from Baseline  LS-Mean (95% CI) | *P*-value | Change from Baseline  LS-Mean (95% CI) | *P*-value | Change from Baseline  LS-Mean (95% CI) | *P*-value |
| --- | --- | --- | --- | --- | --- | --- | --- | --- | --- | --- | --- |
| Non-Surgical hand |  |  |  |  |  |  |  |  |  |  |  |
| Presurgical | 6.66 (0.33) | **Reference** |  |  |  |  |  |  |  |  |  |
| 3 days | 6.35 (0.33) | −0.30 (−0.73 to 0.12) | 0.1621 | **Reference** |  |  |  |  |  |  |  |
| 2 weeks | 6.44 (0.31) | −0.22 (−0.64 to 0.20) | 0.3051 | 0.09 (−0.35 to 0.52) | 0.7008 | **Reference** |  |  |  |  |  |
| 4 weeks | 6.44 (0.34) | −0.22 (−0.53 to 0.09) | 0.1700 | 0.09 (−0.28 to 0.45) | 0.6428 | −0.00 (−0.35 to 0.35) | 1.0000 | **Reference** |  |  |  |
| 16 weeks | 6.29 (0.33) | −0.37 (−0.77 to 0.04) | 0.0792 | −0.06 (−0.53 to 0.41) | 0.8007 | −0.15 (−0.57 to 0.27) | 0.4959 | −0.15 (−0.42 to 0.13) | 0.2935 | **Reference** |  |
| 24 weeks | 6.60 (0.45) | −0.06 (−0.70 to 0.58) | 0.8518 | 0.24 (−0.43 to 0.91) | 0.4757 | 0.16 (−0.58t o 0.89) | 0.6727 | 0.16 (−0.46 to 0.78) | 0.6157 | 0.30 (−0.27 to 0.88) | 0.2949 |
| Presurgical to 24 weeks |  | −0.01 (−0.12 to 0.09) | 0.8019 |  |  |  |  |  |  |  |  |
| Surgical hand |  |  |  |  |  |  |  |  |  |  |  |
| Presurgical | 6.23 (0.32) | **Reference** |  |  |  |  |  |  |  |  |  |
| 3 days | 3.68 (0.29) | −2.55 (−3.08 to −2.01) | <0.0001 | **Reference** |  |  |  |  |  |  |  |
| 2 weeks | 4.73 (0.32) | −1.50 (−1.91 to −1.09) | <0.0001 | 1.05 (0.63 to 1.47) | <0.0001 | **Reference** |  |  |  |  |  |
| 4 weeks | 5.41 (0.32) | −0.82 (−1.17 to −0.46) | <0.0001 | 1.73 (1.27 to 2.19) | <0.0001 | 0.68 (0.44 to 0.92) | <0.0001 | **Reference** |  |  |  |
| 16 weeks | 5.70 (0.34) | −0.54 (−0.98 to −0.09) | 0.0185 | 2.01 (1.43 to 2.60) | <0.0001 | 0.96 (0.56 to 1.37) | <0.0001 | 0.28 (−0.04 to 0.60) | 0.0866 | **Reference** |  |
| 24 weeks | 6.24 (0.55) | 0.01 (−0.84 to 0.87) | 0.9777 | 2.56 (1.45 to 3.67) | <0.0001 | 1.51 (0.55 to 2.48) | 0.0021 | 0.83 (−0.02 to 1.68) | 0.0561 | 0.55 (−0.30 to 1.40) | 0.2037 |
| Presurgical to 24 weeks |  | 0.19 (0.04 to 0.35) | 0.0134 |  |  |  |  |  |  |  |  |
