# Supplementary material for: Novel minimally invasive carpal tunnel release using a specialized surgical kit: a prospective multi-center case series
Source: BMC Musculoskelet Disord. 2025 Apr 8;26:346. doi: 10.1186/s12891-025-08612-0 (PMC11980155; doi:10.1186/s12891-025-08612-0)
Supplement: Supplementary file 1 — Additional file 1 [file 12891_2025_8612_MOESM1_ESM.zip › Supplement Table 4.docx]

Supplement Table 4. Time-course of mean change in Boston Carpal Tunnel Questionnaire from baseline, 3 days, and 2, 4, 16, and 24 weeks

|  | LS-Mean (SE) | Change from Baseline  LS-Mean (95% CI) | *P*-value | Change from Baseline  LS-Mean (95% CI) | *P*-value | Change from Baseline  LS-Mean (95% CI) | *P*-value | Change from Baseline  LS-Mean (95% CI) | *P*-value | Change from Baseline  LS-Mean (95% CI) | *P*-value |
| --- | --- | --- | --- | --- | --- | --- | --- | --- | --- | --- | --- |
| 1. Symptom Severity Scale |  |  |  |  |  |  |  |  |  |  |  |
| Presurgical | 28.17 (1.27) | **Reference** |  |  |  |  |  |  |  |  |  |
| 3 days | 22.83 (1.15) | −5.34 (−8.41 to −2.28) | 0.0006 | **Reference** |  |  |  |  |  |  |  |
| 2 weeks | 18.49 (0.84) | −9.68 (−12.26 to −7.11) | <0.0001 | −4.34 (−5.71 to −2.97) | <0.0001 | **Reference** |  |  |  |  |  |
| 4 weeks | 17.22 (0.94) | −10.95 (−14.13 to −7.77) | <0.0001 | −5.61 (−7.78 to −3.44) | <0.0001 | −1.27 (−2.50 to −0.03) | 0.0439 | **Reference** |  |  |  |
| 16 weeks | 15.78 (1.04) | −12.39 (−15.73 to −9.05) | <0.0001 | −7.05 (−9.35 to −4.75) | <0.0001 | −2.71 (−4.28 to −1.14) | 0.0007 | −1.44 (−2.58 to −0.29) | 0.0138 | **Reference** |  |
| 24 weeks | 14.34 (0.75) | −13.83 (−16.65 to −11.01) | <0.0001 | −8.49 (−10.67 to −6.31) | <0.0001 | −4.15 (−5.57 to −2.73) | <0.0001 | −2.88 (−4.36 to −1.40) | 0.0001 | −1.44 (−3.23 to 0.35) | 0.1154 |
| Presurgical to 24w |  | −2.62 (−3.13 to −2.10) | <0.0001 |  |  |  |  |  |  |  |  |
| 2. Functional Status Scale |  |  |  |  |  |  |  |  |  |  |  |
| Presurgical | 14.20 (0.96) | **Reference** |  |  |  |  |  |  |  |  |  |
| 3 days | 17.68 (1.23) | 3.49 (1.56 to 5.42) | 0.0004 | **Reference** |  |  |  |  |  |  |  |
| 2 weeks | 13.37 (0.92) | −0.83 (−2.29 to 0.64) | 0.2674 | −4.32 (−6.29 to −2.35) | <0.0001 | **Reference** |  |  |  |  |  |
| 4 weeks | 11.98 (0.81) | −2.22 (−3.53 to −0.91) | 0.0009 | −5.71 (−7.49 to −3.93) | <0.0001 | −1.39 (−2.41 to −0.37) | 0.0076 | **Reference** |  |  |  |
| 16 weeks | 10.68 (0.80) | −3.51 (−5.24 to −1.78) | <0.0001 | −7.00 (−9.04 to −4.96) | <0.0001 | −2.68 (−4.03 to −1.34) | <0.0001 | −1.29 (−2.22 to −0.37) | 0.0062 | **Reference** |  |
| 24 weeks | 10.24 (0.73) | −3.95 (−5.66 to −2.24) | <0.0001 | −7.44 (−9.69 to −5.19) | <0.0001 | −3.12 (−4.25 to −1.99) | <0.0001 | −1.73 (−2.75 to −0.71) | 0.0009 | −0.44 (−1.38 to 0.51) | 0.3621 |
| Presurgical to 24 weeks |  | −1.20 (−1.57 to −0.84) | <0.0001 |  |  |  |  |  |  |  |  |

LS-Mean: least squares mean; SE: standard error; CI: confidence intervals.
