# Supplementary material for: Novel minimally invasive carpal tunnel release using a specialized surgical kit: a prospective multi-center case series
Source: BMC Musculoskelet Disord. 2025 Apr 8;26:346. doi: 10.1186/s12891-025-08612-0 (PMC11980155; doi:10.1186/s12891-025-08612-0)
Supplement: Supplementary file 1 — Additional file 1 [file 12891_2025_8612_MOESM1_ESM.zip › Supplement Table 5.docx]

Supplement Table 5. Time course of average change in results from baseline, 3 days, and 2, 4, 16, and 24 weeks

|  | N | Surgical hand | Normal hand | P1 | P2 |
| --- | --- | --- | --- | --- | --- |
| Boston Carpal Tunnel Questionnaire-Symptom Severity Scale |  |  |  |  |  |
| Presurgical | 41 | 28.17 (8.25) |  |  |  |
| 3d | 41 | 22.83 (7.46) |  |  |  |
| 2w | 41 | 18.49 (5.42) |  |  |  |
| 4w | 41 | 17.22 (6.09) |  |  |  |
| 16w | 32 | 15.78 (6.72) |  |  |  |
| 24w | 37 | 14.34 (4.87) |  |  |  |
| Boston Carpal Tunnel Questionnaire-  Functional Status Scale |  |  |  |  |  |
| Presurgical | 41 | 14.20 (6.25) |  |  |  |
| 3d | 41 | 17.68 (7.94) |  |  |  |
| 2w | 41 | 13.37 (5.99) |  |  |  |
| 4w | 41 | 11.98 (5.24) |  |  |  |
| 16w | 32 | 10.68 (5.16) |  |  |  |
| 24w | 37 | 10.24 (4.75) |  |  |  |
| Grip strength(kg) |  |  |  |  |  |
| Presurgical |  | 19.85 (10.58) | 22.59 (10.60) | 0.2462 | 0.2201 |
| 3d | 41 | 7.79 (6.69) | 22.00 (10.36) | <0.0001 | <0.0001 |
| 2w | 41 | 13.8 (8.84) | 22.15 (7.92) | <0.0001 | <0.0001 |
| 4w | 41 | 16.85 (10.41) | 22.56 (9.49) | 0.0113 | 0.0019 |
| 16w | 41 | 19.83 (11.01) | 24.20 (9.99) | 0.0637 | 0.0323 |
| 24w | 32 | 22.34 (11.37) | 25.71 (10.4) | 0.1659 | 0.0774 |
| Pinch strength(kg) |  |  |  |  |  |
| Presurgical | 41 | 6.23 (2.09) | 6.66 (2.16) | 0.3669 | 0.3876 |
| 3d | 41 | 3.68 (1.87) | 6.35 (2.13) | <0.0001 | <0.0001 |
| 2w | 41 | 4.73 (2.09) | 6.44 (1.98) | 0.0003 | 0.0004 |
| 4w | 41 | 5.41 (2.07) | 6.44 (2.17) | 0.0317 | 0.0234 |
| 16w | 32 | 5.70 (2.18) | 6.29 (2.15) | 0.2150 | 0.2022 |
| 24w | 37 | 6.24 (3.54) | 6.60 (2.95) | 0.6241 | 0.2697 |
| Visual Analog Scale (VAS) |  |  |  |  |  |
| Presurgical | 41 | 3.24 (2.96) |  |  |  |
| 3d | 41 | 2.51 (2.68) |  |  |  |
| 2w | 41 | 1.51 (1.82) |  |  |  |
| 4w | 41 | 1.24 (1.81) |  |  |  |
| 16w | 32 | 0.59 (1.50) |  |  |  |
| 24w | 37 | 0.46 (1.07) |  |  |  |
| Nerve conduction velocity (NCV) |  |  |  |  |  |
| Latency(ms) |  |  |  |  |  |
| Presurgical | 41 | 4.63 (1.16) | 3.98 (1.10) | 0.0129 | 0.0039 |
| 24w | 34 | 4.05 (1.06) | 3.94 (1.00) | 0.6146 | 0.4144 |
| Velocity (m/s) |  |  |  |  |  |
| Presurgical | 41 | 32.91 (8.25) | 39.32 (9.54) | 0.0022 | 0.0023 |
| 24w | 34 | 38.70 (8.99) | 40.62 (9.16) | 0.3550 | 0.4094 |

Values are mean (SD), median (interquartile range, IQR(Q1, 25% and Q3, 75%) or percentage (%).

The mean differences were estimated by using the t test (p1) or the Wilcoxon Rank Sum test (p2)
